# Effect of MTA and Portland Cement on Fracture Resistance of Dentin

**DOI:** 10.5681/joddd.2013.014

**Published:** 2013-05-30

**Authors:** Maryam Forghani, Maryam Bidar, Fatemeh Shahrami, Mahmoud Bagheri, Maryam Mohammadi, Niloufar Attaran Mashhadi

**Affiliations:** ^1^Assistant Professor of Endodontics, Dental Material Research Center, School of Dentistry, Mashhad University of Medical Sciences, Mashhad, Iran; ^2^Professor of Endodontics, Dental Research Center, School of Dentistry, Mashhad University of Medical Sciences, Mashhad, Iran; ^3^Endodontist, Private Practice, Mashhad, Iran; ^4^General Practitioner, School of Dentistry, Mashhad University of Medical Sciences, Mashhad, Iran; ^5^General Practitioner, Private Practice, Mashhad, Iran; ^6^Dental Student, Gilan University of Medical Sciences, Rasht, Iran

**Keywords:** Dentin, fracture resistance, mineral trioxide aggregate, Portland cement

## Abstract

***Background and aims.*** It is important to evaluate the effects of endodontic materials on tooth structures to avoid endodontic treatment failure. The aim of the present study was to investigate the effect of mineral trioxide aggregates (MTA) and Portland cement (PC) on fracture resistance of dentin.

***Materials and methods.*** Thirty-six freshly extracted human single-rooted premolar teeth were selected. The crowns were removed and the roots were randomly divided into two experimental groups and one control group. The root samples were longitudinally divided into two halves and a dentin bar (2×2×10 mm) was cut from each root section for short-term (2weeks) and long-term (12 weeks) evaluations. The root sections in the experimental groups were exposed to MTA or PC, while keeping the control group specimens in physiologic saline. The fracture resistance of each specimen was measured using an Instron testing machine. The results were statistically analyzed using ANOVA, a post hoc Tukey test and paired t-test at 5% significance level.

***Results.*** The fracture resistance of MTA-treated specimens significantly increased between 2 and 12 weeks (P<0.05). After 12 weeks, MTA-treated specimens had the highest fracture resistance. In the PC group, the fracture resistance of specimens did not change significantly over time (P>0.05).

***Conclusion.*** The results showed that MTA increased the fracture resistance of root dentin, while PC had no significant effect on dentin fracture resistance.

## Introduction

Calcium hydroxide [Ca(OH)_2_] has been advocated as an intracanal medicament in the treatment of inflammatory external root resorption ^1,2^ and for induction of apical closure in nonvital immature teeth.^3^ However, these treatments may continue for several months before the desired effects are achieved.^4,5^ It has been proposed that long-term exposure to Ca(OH)_2_ will lead to weakening of roots and increased susceptibility to fracture either spontaneously or due to minor impacts.^6,7^

Mineral trioxide aggregate (MTA), a Portland cement-based material, has many endodontic applications and has largely replaced Ca(OH)_2_.^8,9^ MTA’s suitability as a material for use in the treatment of non-vital immature teeth has been investigated. As with Ca(OH)_2_, it shows high alkalinity when it is freshly mixed.^10^ It has been reported that MTA induces apical hard tissue formation at a rate similar to that seen with Ca(OH)_2_.^11^ Its application in the treatment of external infection-related root resorption has also been described in some cases.^12-14^

In terms of fracture resistance, MTA has shown similar^15^ or better results^16-18^ compared to Ca(OH)_2_. MTA use might mitigate the potential weakening of roots associated with long-term Ca(OH)_2_ treatment.^19^ Despite its advantages, MTA has some limitations, including long setting time, inadequate compressive strength, difficult handling and high cost.^20,21^

The principal ingredients and the amount of arsenic in Portland cement (PC),^22-25^ as well as its biocompatibility^26-28^ and physicochemical behavior,^29^ are similar to those of MTA. The setting time of PC can be reduced by removing gypsum from manufacturing process without affecting other properties.^30^ Shahi et al^31^ compared the sealing ability of MTA and PC as a root-end filling material and concluded that these two materials have similar microleakage. Gomes Cornélio et al^32^ reported that a major drawback of Portland cement is its lack of radiopacity, and evaluated the cytotoxic effect of three different radiopacifying agents associated with PC. Their results showed that Portland cement containing bismuth oxide, zirconium oxide or calcium tungstate is not cytotoxic. Zeferino et al^33^ also reported that Portland cement containing 15% bismuth oxide is not genotoxic and cytotoxic. Therefore, PC can be considered a possible substitute for MTA because of its similar properties and lower cost.

Although Portland cement can be applied for endodontic treatment, no study has ever evaluated its effect on fracture resistance of dentin. The aim of this study was to evaluate the effect of MTA and PC on the fracture resistance of root dentin.

## Materials and Methods

This in vitro study model was based on the method described by White et al,^15^ with some modifications. Thirty-six freshly extracted, single-rooted human premolar teeth without decay, crack or fracture were used for the experiment. The teeth were stored in physiologic saline after extraction to prevent dehydration. They were randomly assigned to two experimental groups and one control group (n=12). The crowns were removed at the level of cemento-enamel junction (CEJ), using a high-speed fissure bur. The root samples were longitudinally divided into two halves using a disk saw (Edmta Golden, S.A.W, and Swiss) and a dentin bar (2×2×10 mm) was cut from each root section for short-term (2 weeks) and long-term (12 weeks) evaluations.

Pro-Root MTA (Dentsply-Tulsa Dental, Tulsa, OK, USA) and Portland cement (Tehran Cement Co., Tehran, Iran) were mixed with distilled water using a 3:1 powder-liquid ratio to achieve a putty consistency and placed in 2×2×10 mm molds. Root sections were then placed in contact with MTA or PC. Only one side of each dentinal bar was exposed to the material to simulate contact of the material with root dentin in a clinical situation. The specimens in the control group were placed in Petri dishes containing physiologic saline. The samples were then incubated at 37°C and 100% humidity.

After completion of the evaluation period, each sample was rinsed with saline and mounted in self-cured acrylic resin in a manner to protrude exactly 6 mm of the block. The fracture resistance was measured with a universal testing machine (Instron Crop., Canton, MA, USA) at a crosshead speed of 1 mm/min. A compressive force was applied at a point 2 mm from the acrylic base using a chisel-shaped tip. Maximum force required to fracture each specimen was recorded. The results were statistically analyzed using ANOVA, a post hoc Tukey test and paired t-test at 5% significance level.

## Results

The means and standard deviations of the fracture resistance calculated from the raw data are presented in Table 1.

**Table 1:** Means and standard deviations of the facture resistance of samples at two evaluation periods

	Evaluation Period		P-value
Group	2 weeks	12 weeks	
MTA	106.1±37.3	156.1±19.1	P<0.05
Portland	97.1±29.4	94.2±20.2	P>0.05
Saline	98.1±31.8	82.8±12.2	P>0.05

P>0.05 (within groups).

Two-way ANOVA demonstrated a statistically significant interaction between the type of the material and exposure time (P<0.05). Therefore, the materials were compared in each of the two time periods using one-way ANOVA and a post hoc Tukey test.

MTA-treated specimens had the highest fracture resistance at 2-week interval (106.1 N). However, there were no significant differences between the groups (P>0.05).

After 12 weeks, the force required to fracture dentin in the MTA-treated specimens was significantly higher than the other two groups (P<0.05). There were no significant differences between the PC and control groups (P>0.05).

Paired t-test demonstrated a significant increase in fracture resistance of MTA-treated specimens between 2- and 12-week periods (P<0.05). In the PC and control groups the fracture resistance of dentin did not change significantly after 12 weeks (P>0.05).

## Discussion

Many previous studies have utilized bovine or sheep teeth to evaluate the fracture resistance of dentin.^6,15-17^ However, human premolar teeth were used in the present study. Dentinal bars with the same size and shape were used to reduce the effect of variability associated with different dentinal wall thickness in human teeth. It was assumed that if any of the tested materials affected the structural integrity of human dentin, it would have likely presented itself in the value of compressive forces required to fracture the dentin bars.

In this study model, hardening of MTA and PC after setting might have influenced the fracture resistance of the samples. However, this reinforcement effect can be seen in situations in which the root canals have been obturated with these materials.^34,35^

After 12 weeks, the fracture resistance of dentin exposed to MTA was significantly higher than that in the other groups (P<0.05). The ability of MTA to strengthen tooth structure in previous studies has yielded controversial results. White et al^15^ showed weakening of the tooth structure after 5 weeks of exposure to MTA. They believed that breakdown of the protein structure by the alkalinity of MTA was responsible for this result. However, Andreasen et al^16^ reported that fracture resistance of teeth treated with MTA was higher than those filled with either saline or Ca(OH)_2_, although the small sample size of the study resulted in statistically insignificant differences. Other ex vivo studies evaluating fracture resistance of immature teeth filled with MTA^18,34,35^have demonstrated reinforcing effect of MTA. Milani et al^35^ attributed this finding to similar elastic modulus of MTA and dentin. A finite element analysis showed that a material with elastic modulus close to that of dentin can reinforce the weakened root.^36^

It has been reported that degradation of dentinal organic matrix is mainly mediated by matrix metalloproteinases (MMPs).^37^,^38^ Hatibović-Kofman et al^17^ evaluated fracture resistance of teeth treated with Ca(OH)_2_ and MTA. They reported that tissue inhibitor of MMPs was expressed in MTA-treated dentin, but it was undetectable in dentin treated with Ca(OH)_2_. They concluded that increase in fracture resistance in the MTA group may be related to this finding. Tomson et al^39^ demonstrated that MTA can release the bioactive molecules that have been sequestered within dentin matrix. It was thought that the change in the dentin matrix as a result of a biological interaction between MTA and dentin may inhibit destruction of the organic matrix of dentin.

For the PC-treated specimens, the mean fracture resistance did not change significantly over time (97.1 N and 94.2 N after 2 and 12 weeks, respectively). After 12 weeks, the fracture resistance of dentin treated with PC was similar to the control group.

The differences in the results could be explained by the fact that MTA and PC are not identical materials. MTA has significantly smaller particles^40,41^ and undergoes a better purification process than PC.^40^ Several previous studies have also shown better results for MTA compared to PC, such as higher resistance to displacement^42^ and better efficacy in promoting the biomineralization process.^43^

Since the effect of MTA on the fracture resistance of dentin is time-dependent,^17^ further studies with longer time intervals are suggested. Histological analysis of the affected dentin can also help explain the mechanisms affecting fracture resistance of dentin.

## Conclusion

Within the limitations of this study it was concluded that MTA can increase the fracture resistance of root dentin over time, while PC has no significant effect on dentin fracture resistance.

## Acknowledgement

The results presented in this study have been taken from a student thesis (no: 2093) and also financially supported by the Vice Chancellor for Research of Mashhad University of Medical Sciences.

## References

[1] Tronstad L, Andreasen JO, Hasselgren G, Kristerson L, Riis I (1981). PH changes in dental tissues after root canal filling with calcium hydroxide. J Endod.

[2] Fuss Z, Tsesis I, Lin S (2003). Root resorption--diagnosis, classification and treatment choices based on stimulation factors. Dent Traumatol.

[3] Fava LR (1994). A clinical evaluation of one and two-appointment root canal therapy using calcium hydroxide. Int Endod J.

[4] Trope M (1995). Clinical management of the avulsed tooth. Dent Clin North Am.

[5] Sheehy EC, Roberts GJ (1997). Use of calcium hydroxide for apical barrier formation and healing in non-vital immature permanent teeth: a review. Br Dent J.

[6] Andreasen JO, Farik B, Munksgaard EC (2002). Long-term calcium hydroxide as a root canal dressing may increase risk of root fracture. Dent Traumatol.

[7] Nerwich A, Figdor D, Messer HH (1993). pH changes in root dentin over a 4-week period following root canal dressing with calcium hydroxide. J Endod.

[8] Bogen G, Kuttler S (2009). Mineral trioxide aggregate obturation: a review and case series. J Endod.

[9] Parirokh M, Torabinejad M (2010). Mineral trioxide aggregate: a comprehensive literature review--Part III: Clinical applications, drawbacks, and mechanism of action. J Endod.

[10] Torabinejad M, Hong CU, McDonald F, Pitt Ford TR (1995). Physical and chemical properties of a new root-end filling material. J Endod.

[11] Shabahang S, Torabinejad M, Boyne PP, Abedi H, McMillan P (1999). A comparative study of root-end induction using osteogenic protein-1, calcium hydroxide, and mineral trioxide aggregate in dogs. J Endod.

[12] Karp J, Bryk J, Menke E, McTigue D (2006). The complete endodontic obturation of an avulsed immature permanent incisor with mineral trioxide aggregate: a case report. Pediatr Dent.

[13] Aggarwal V, Singla M (2010). Management of inflammatory root resorption using MTA obturation - a four year follow up. Br Dent J.

[14] Oliveira TM, Sakai VT, Silva TC, Santos CF, Abdo RC, Machado MA (2008). Mineral trioxide aggregate as an alternative treatment for intruded permanent teeth with root resorption and incomplete apex formation. Dent Traumatol.

[15] White JD, Lacefield WR, Chavers LS, Eleazer PD (2002). The effect of three commonly used endodontic materials on the strength and hardness of root dentin. J Endod.

[16] Andreasen JO, Munksgaard EC, Bakland LK (2006). Comparison of fracture resistance in root canals of immature sheep teeth after filling with calcium hydroxide or MTA. Dent Traumatol.

[17] Hatibović-Kofman S, Raimundo L, Zheng L, Chong L, Friedman M, Andreasen JO (2008). Fracture resistance and histological findings of immature teeth treated with mineral trioxide aggregate. Dent Traumatol.

[18] Tuna EB, Dinçol ME, Gençay K, Aktören O (2011). Fracture resistance of immature teeth filled with BioAggregate, mineral trioxide aggregate and calcium hydroxide. Dent Traumatol.

[19] Hansen SW, Marshall JG, Sedgley CM (2011). Comparison of intracanal EndoSequence Root Repair Material and ProRoot MTA to induce pH changes in simulated root resorption defects over 4 weeks in matched pairs of human teeth. J Endod.

[20] Mooney GC, North S (2008). The current opinions and use of MTA for apical barrier formation of non-vital immature permanent incisors by consultants in paediatric dentistry in the UK. Dent Traumatol.

[21] Camilleri J (2009). Evaluation of selected properties of mineral trioxide aggregate sealer cement. J Endod.

[22] Camilleri J (2008). The chemical composition of mineral trioxide aggregate. J Conserv Dent.

[23] Asgary S, Eghbal MJ, Parirokh M, Ghoddusi J, Kheirieh S, Brink F (2009). Comparison of mineral trioxide aggregate's composition with Portland cements and a new endodontic cement. J Endod.

[24] Schembri M, Peplow G, Camilleri J (2010). Analyses of heavy metals in mineral trioxide aggregate and Portland cement. J Endod.

[25] De-Deus G, de Souza  MC, Sergio Fidel  RA, Fidel SR, de Campos  RC, Luna AS (2009). Negligible expression of arsenic in some commercially available brands of Portland cement and mineral trioxide aggregate. J Endod.

[26] Holland R, de Souza V, Nery MJ, Faraco Júnior  IM, Bernabé PF, Otoboni Filho  JA (2001). Reaction of rat connective tissue to implanted dentin tube filled with mineral trioxide aggregate, Portland cement or calcium hydroxide. Braz Dent J.

[27] Saidon J, He J, Zhu Q, Safavi K, Spångberg LS (2003). Cell and tissue reactions to mineral trioxide aggregate and Portland cement. Oral Surg Oral Med Oral Pathol Oral Radiol Endod.

[28] Menezes R, Bramante CM, Letra A, Carvalho VG, Garcia RB (2004). Histologic evaluation of pulpotomies in dog using two types of mineral trioxide aggregate and regular and white Portland cements as wound dressings. Oral Surg Oral Med Oral Pathol Oral Radiol Endod.

[29] Gonçalves JL, Viapiana R, Miranda CE, Borges AH, Cruz Filho AM (2010). Evaluation of physico-chemical properties of Portland cements and MTA. Braz Oral Res.

[30] Camilleri J (2008). The physical properties of accelerated Portland cement for endodontic use. Int Endod J.

[31] Shahi S, Yavari HR, Rahimi S, Eskandarinezhad M, Shakouei S, Unchi M (2011). Comparison of the sealing ability of mineral trioxide aggregate and Portland cement used as root-end filling materials. J Oral Sci.

[32] Gomes Cornélio AL, Salles LP, Campos da Paz M, Cirelli JA, Guerreiro-Tanomaru JM, Tanomaru Filho  M (2011). Cytotoxicity of Portland cement with different radiopacifying agents: a cell death study. J Endod.

[33] Zeferino EG, Bueno CE, Oyama LM, Ribeiro DA (2010). Ex vivo assessment of genotoxicity and cytotoxicity in murine fibroblasts exposed to white MTA or white Portland cement with 15% bismuth oxide. Int Endod J.

[34] Bortoluzzi EA, Souza EM, Reis JM, Esberard RM, Tanomaru-Filho M (2007). Fracture strength of bovine incisors after intra-radicular treatment with MTA in an experimental immature tooth model. Int Endod J.

[35] Milani AS, Rahimi S, Borna Z, Jafarabadi MA, Bahari M, Deljavan AS (2012). Fracture resistance of immature teeth filled with mineral trioxide aggregate or calcium-enriched mixture cement: An ex vivo study. Dent Res J (Isfahan).

[36] Li LL, Wang ZY, Bai ZC, Mao Y, Gao B, Xin HT, et al. Three-dimensional finite element analysis of weakened roots restored with different cements in combination with titanium alloy posts. Chin Med J (Engl) 2006; 20;119:305-11. 16537026

[37] Tjäderhane L, Larjava H, Sorsa T, Uitto VJ, Larmas M, Salo T (1998). The activation and function of host matrix metalloproteinases in dentin matrix breakdown in caries lesions. J Dent Res.

[38] Chaussain-Miller C, Fioretti F, Goldberg M, Menashi S. The role of matrix metalloproteinases (MMPs) in human caries. J Dent Res 2006;85:22-32. Review. 10.1177/154405910608500104 16373676

[39] Tomson PL, Grover LM, Lumley PJ, Sloan AJ, Smith AJ, Cooper PR (2007). Dissolution of bio-active dentine matrix components by mineral trioxide aggregate. J Dent.

[40] Dammaschke T, Gerth HU, Züchner H, Schäfer E (2005). Chemical and physical surface and bulk material characterization of white ProRoot MTA and two Portland cements. Dent Mater.

[41] Hwang YC, Kim DH, Hwang IN, Song SJ, Park YJ, Koh JT (2011). Chemical constitution, physical properties, and biocompatibility of experimentally manufactured Portland cement. J Endod.

[42] Vosoughhosseini S, Lotfi M, Shahi S, Baloo H, Mesgariabbasi M, Saghiri MA et al. Influence of white versus gray mineral trioxide aggregate on inflammatory cells. J Endod 2008;34:715-7. 10.1016/j.joen.2008.03.005 18498897

[43] Shahi S, Rahimi S, Yavari HR, Mokhtari H, Roshangar L, Abasi MM (2010). Effect of mineral trioxide aggregates and Portland cements on inflammatory cells. J Endod.

